# Lexical access in sign language: a computational model

**DOI:** 10.3389/fpsyg.2014.00428

**Published:** 2014-05-15

**Authors:** Naomi K. Caselli, Ariel M. Cohen-Goldberg

**Affiliations:** Department of Psychology, Tufts UniversityMedford, MA, USA

**Keywords:** neighborhood density, sign language, spreading activation, sub-lexical processing, sign perception, speech perception, lexical access

## Abstract

Psycholinguistic theories have predominantly been built upon data from spoken language, which leaves open the question: How many of the conclusions truly reflect language-general principles as opposed to modality-specific ones? We take a step toward answering this question in the domain of lexical access in recognition by asking whether a single cognitive architecture might explain diverse behavioral patterns in signed and spoken language. Chen and Mirman (2012) presented a computational model of word processing that unified opposite effects of neighborhood density in speech production, perception, and written word recognition. Neighborhood density effects in sign language also vary depending on whether the neighbors share the same handshape or location. We present a spreading activation architecture that borrows the principles proposed by Chen and Mirman (2012), and show that if this architecture is elaborated to incorporate relatively minor facts about either (1) the time course of sign perception or (2) the frequency of sub-lexical units in sign languages, it produces data that match the experimental findings from sign languages. This work serves as a proof of concept that a single cognitive architecture could underlie both sign and word recognition.

## Introduction

One of the most important discoveries about language in the past half-century is arguably the fact that signed and spoken languages share fundamental aspects of their linguistic structure (Klima and Bellugi, 1979; Wilbur, 1979; Poizner et al., 1987; Lucas and Valli, 1992; Emmorey, 2002; Sandler and Lillo-Martin, 2006). The fact that all natural languages have common grammatical principles despite vast differences in modality has had critical implications for theories of the human language faculty and its evolution (e.g., Pinker, 1994; Jackendoff, 2002). Though a parallel line of research exists comparing the psycholinguistic mechanisms of signed and spoken language (Petitto et al., 2000; Sandler and Lillo-Martin, 2006; Emmorey et al., 2007; MacSweeney et al., 2008; Berent et al., 2013), much work remains. Far less is known, for example, about whether the mental lexicon is organized similarly across modalities and whether words and signs are activated and selected in similar ways. In the same way that the discovery of a common set of grammatical principles influenced theories of universal grammar, discovering similarities (or differences) in processing can profoundly advance our knowledge about psycholinguistic systems.

Within the psycholinguistic framework, the comprehension of a single word ultimately involves mapping a physical signal onto its meaning while the production of a single word involves the reverse process, mapping meaning to a physical signal. Multiple stages of processing have been posited to take place in between these two endpoints, most generally the identification (or in production, the preparation) of sub-lexical and lexical units (e.g., Dell, 1986; McClelland and Elman, 1986). According to a number of accounts, signed and spoken languages, should have similarly organized semantic systems (e.g., Jackendoff, 2012). At the same time, their most peripheral elements clearly differ: signed languages utilize manual and facial articulators and are perceived through the visual system while spoken languages are produced with the oral articulators and are perceived through the auditory system.

There are a number of ways the language processing architecture could be organized with respect to these facts about the signed and spoken modalities. On the one hand, it's possible that signed and spoken languages utilize different cognitive mechanisms for all but the most central (i.e., semantic) stages of processing. It is also reasonable that a continuum of processing similarity could exist, where signed and spoken languages utilize similar cognitive mechanisms to achieve semantic processing but rely on increasingly different mechanisms to access the lexicon and process sub-lexical elements. Finally, it is also possible that identical psycholinguistic mechanisms underlie all stages of processing, with only the specific content differing across modalities (e.g., manual sign location vs. oral place of articulation).

In the present paper we consider the cognitive processes that underlie word and sign retrieval, that is, the mechanisms responsible for *lexical access*. We review the literature and find evidence that sign retrieval is influenced by factors that are specific to signed languages, suggesting that there may be modality-specific mechanisms for retrieving words and signs from the mental lexicon. Using a computational model, we explore the possibility that these differences are in fact superficial and that a common mechanism underlies lexical access in both modalities.

Computational modeling is a useful tool in the development of cognitive theories. In such an investigation, the modeler instantiates a particular cognitive theory in the code of a computer program. This encoding process is beneficial in and of itself because it requires the modeler to state the theory in computationally explicit terms, defining its properties precisely. Once the theory has been translated thusly, the modeler may then use the program to test the theory. By running the program, the modeler runs a simulation of the theory, obtaining specific outputs for specific inputs. This allows the modeler to determine the predictions of the theory (e.g., in lexical access, if a sign's basic components are activated in this sequence, what are the consequences for the sign's activation?). This can be especially important in complex systems where it may be otherwise difficult to determine how the system will function (e.g., how are signs activated in a system with many connections and feedback loops?). Finally, the modeler compares the predictions generated by the simulation to empirical data. To the extent that the behavior of the simulation matches human behavior, we can conclude that the principles that underlie human behavior might be the same as those that underlie the model (see McCloskey, 1991, for a discussion of the difficulties in assigning credit and blame in simulations). Failure to capture empirical performance, by contrast, would provide an argument that the theory instantiated by the computer program is not an accurate description of human cognition (e.g., Goldberg and Rapp, 2008). Like laboratory experiments, most simulation work focuses on explicating a particular aspect of a cognitive domain. In this pursuit, simulations typically systematically vary the property of interest while keeping extraneous factors constant, either by using constant values or by not modeling the property at all. The advantage of this approach in modeling and in laboratory experiments is that it is possible to isolate the effects of variables of interest, though it reduces the ecological validity of the study. Nevertheless, simulation can form an important role in the feedback loop of theory building (Peschl and Scheutz, 2001).

In the present paper, we develop a computational simulation of sign access that imports core access principles that were developed specifically to account for phenomena observed in spoken (and written) lexical access (Chen and Mirman, 2012). The strength of this model in the present case is that it contains no elements that are specific to signed or spoken languages, allowing us to determine if an abstract set of principles is capable of accounting for lexical access across modalities. We show that if a model containing these core principles is elaborated to incorporate relatively minor facts about either (1) the time course of sign perception or (2) the frequency of sub-lexical units in sign languages, it produces data that qualitatively match the experimental findings from sign languages. We argue that these simulations serve as an existence proof, demonstrating that a single computational mechanism could in theory be responsible for lexical access in signed and spoken languages. Finally, we use the simulation to generate a novel prediction about how lexical access is accomplished in sign language that we hope spurs future research.

In spoken word processing, one of the most well-documented findings is that the degree to which a word is phonologically related to other words influences how that word is processed. In spoken and written language, neighborhood density, a measure of how interconnected a given word is, has been typically been defined as the number of words that differ from the target word by one grapheme or phoneme (Coltheart et al., 1977; Luce and Pisoni, 1998). Psycholinguistic research has demonstrated that neighborhood density influences speech perception, speech production, and written word perception, but the effect differs by task and modality. In spoken production neighborhood density is facilitatory (Vitevitch, 1997, 2002; Mirman et al., 2010 though recent studies have suggested a more complicated picture: Mirman and Graziano, 2013; Sadat et al., 2014) while in spoken perception neighborhood density is inhibitory (e.g., Goldinger et al., 1989; Dufour and Peereman, 2003). In visual word recognition neighborhood density is facilitatory (Andrews, 1992), except for high frequency words in which case neighborhood density is inhibitory (e.g., Grainger et al., 1989; Davis et al., 2009)^1^.

**Figure 1:**
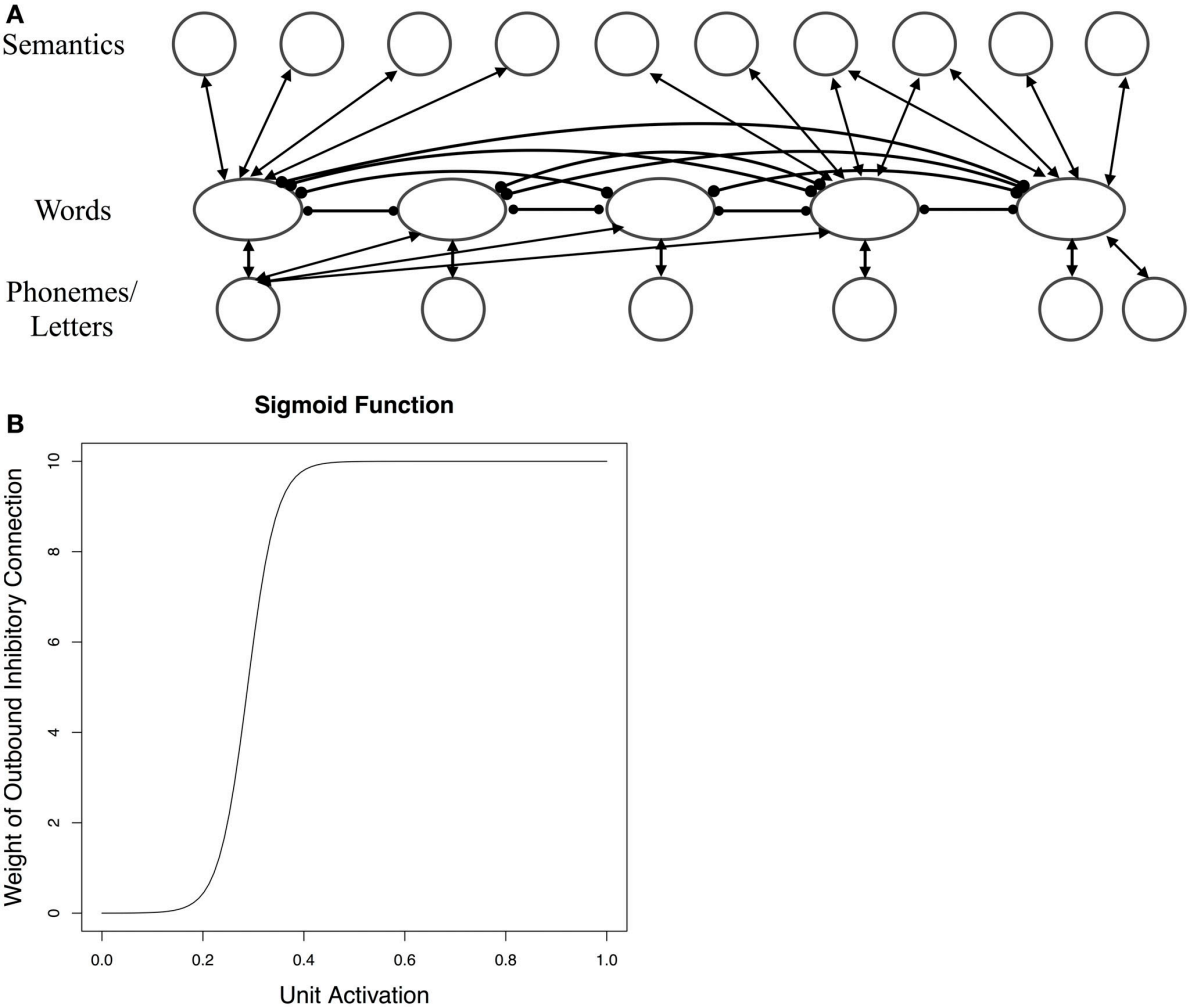
**Chen and Mirman (2012) Architecture**. Panel **(A)** illustrates the spreading activation architecture used by Chen and Mirman (2012) to account for the pattern of reversals of neighborhood density effects in spoken and written language. Facilitatory connections are drawn with arrows, and inhibitory connections are drawn with circle endpoints. In this architecture, as demonstrated in panel **(B)**, the amount of inhibition a given lexical item exerts is scaled by a sigmoid function of its activation. Figures adapted from Chen and Mirman (2012).

Until recently, the theoretical accounts of these neighborhood density effects have differed depending on the modality. For example, in speech perception neighbors were posited to be inhibitory because multiple candidate words compete for selection (McClelland and Elman, 1986), while in speech production neighbors were thought to be facilitatory because of the dominant influence of feedback connections (Dell and Gordon, 2003). Chen and Mirman (2012) proposed a single architecture that attempts to unify the pattern of reversals in *spoken and written language*. At the heart of their architecture is a spreading activation system with two kinds of connections between linguistic units: inhibitory lateral connections between lexical items and facilitatory “vertical” connections between lexical items and phonemes/graphemes and between lexical items and semantic units (see Figure 1A). Vertical connections are bidirectional, allowing for the feedforward as well as feedback flow of activation, while lateral connections are unidirectional, meaning that two lexical items can inhibit each other with different strengths. The system differs from a standard spreading activation architecture in that the strength of a lexical unit's inhibitory connections to other units varies as a function of the unit's activation. Rather than being fixed, inhibitory weights vary according to a sigmoid function: if the unit's activation is low the weight on the inhibitory connection is small; if the unit's activation is high the weight is large (see Figure 1B).

Lexical items thus send both facilitatory *and* inhibitory activation to other lexical items. For example, imagine an individual hears the word *cat*. As phonetic information is translated to phonological information, the matching sub-lexical units /k/, /æ/, and /t/ become active. As sub-lexical units receive activation, they each send activation through feedforward connections to the target word and its neighbors (*cap*, *sat*, *cot*, etc.). As the lexical items become active, they feed activation back to the sub-lexical units, which in turn feed activation forward, facilitating the target and its neighbors. At the same time, as the target and neighbors become active they inhibit each other through lateral (lexical-lexical) connections. Neighbors thus simultaneously activate and inhibit the target word.

Chen and Mirman suggest that the reversals in the direction of neighborhood density effects observed in spoken and written language result not from architectural differences across modalities but from delicate shifts in the balance between the facilitation and inhibition sent by a word's neighbors. When a neighbor is strongly activated, the amount of inhibition it sends outweighs the amount of facilitation it sends, due to the activation-dependent weighting of the inhibitory connections (high activation results in a large inhibitory weight). The net effect on the target item is inhibition. Conversely, when a lexical item is weakly activated, the amount of facilitation it sends outweighs the inhibition, resulting in facilitation of the target word. To generalize, strong neighbors inhibit while weak neighbors facilitate. According to their argument, differences in the task being performed lead to shifts in net facilitation or inhibition, causing neighbors to inhibit spoken recognition but facilitate spoken production. Specifically, neighbors become highly activated during speech perception (and thus have an inhibitory influence) since they are directly activated by sub-lexical units (/k/ /æ/ activate both *cat* and *cap*). By contrast, neighbors are relatively weak in production since the only activation they receive is through feedback from sub-lexical units (*cat* sends feedback activation to /k/ and /æ/, which in turn activate *cap*).

Turning to signed language, sign processing in many ways is like word processing. Like words, signs are accessed automatically (Dupuis and Berent, 2013). Phonological structure is one of the core organizing properties of all languages, including sign languages (Goldin-Meadow et al., 1995). Like the sounds in words, signs are composed of discrete meaningless formal units such as hand configuration or location^2^. As in spoken language, lexical access in signed language is thought to entail a two-step procedure involving sub-lexical and lexical levels of processing in production (Thompson et al., 2005; Corina and Knapp, 2006a; Baus et al., 2008) and perception (Corina and Emmorey, 1993; Corina and Hildebrandt, 2002; Mayberry and Witcher, 2005; Dye and Shih, 2006; Carreiras et al., 2008; Carreiras, 2010).

Far fewer studies have examined the role of “phonological” (formal) neighbors in sign language, though the emerging pattern is that neighbors also influence sign processing. To date, neighbors in sign language have generally been defined differently than they have been defined in spoken language. Rather than defining neighbors as signs that *differ* by one sub-lexical unit (minimal pair neighbors), neighbors have been defined as signs that *share* one sub-lexical unit (though other definitions have also been used: Mayberry and Witcher, 2005; Corina and Knapp, 2006a; Dye and Shih, 2006). Signs that share the same handshape are typically referred to as “handshape neighbors,” signs that share the same location are called “location neighbors,” and so on. Though this approach makes comparison between signed and spoken language somewhat difficult, it has been used in part because there are far fewer minimal pairs in sign languages relative to spoken languages (van der Kooij, 2002).

This approach has revealed that the effect of neighborhood density in sign perception differs depending on the *specific type* of neighbor. In a study of Spanish Sign Language (LSE) processing, Carreiras et al. (2008) found that signs with many *handshape* neighbors (having “dense handshape neighborhoods”) are easier to identify in a lexical decision task than signs with few handshape neighbors. Meanwhile, signs with dense *location* neighborhoods are harder to identify than signs with few location neighbors. Inhibitory effects have also been observed in primed lexical decision tasks in American Sign Language (ASL), where location primes inhibit target processing (Corina and Emmorey, 1993; Corina and Hildebrandt, 2002)^3^. Finally, a similar pattern has been observed in production. In a picture-sign interference task, Catalan Sign Language (LSC) signers named pictures more slowly when the to-be-named picture was presented alongside a distracter sign that used the same location and more quickly when the distracter shared the same handshape or movement (Baus et al., 2008).

It is important to note that these effects have not been universally found. Some studies have failed to find priming effects with either handshape neighbors (Corina and Emmorey, 1993; Dye and Shih, 2006) or location neighbors (Dye and Shih, 2006)^4^ though there is some suggestion that these null effects may be due to varying ISI and insufficient power (see Carreiras, 2010). Similar null effects of location neighbors and handshape neighbors have been documented in production as well (Corina and Knapp, 2006a). There is also some evidence that the effects of neighbors may be modulated by language experience. In the only known study to define neighbors in the same way as spoken language, Mayberry and Witcher (2005) found facilitatory neighborhood effects for signers who started learning ASL between ages 4 and 8, inhibitory effects for signers who started learning ASL between the ages of 9 and 13, and no effects for signers who learned ASL from birth. Clearly more research is needed but to summarize, when neighbors have been defined as signs that share one feature with the target, the studies that have found significant effects have consistently indicated that location neighbors inhibit lexical access while handshape neighbors facilitate access.

Putting these findings together, we see that in spoken language it is the specific task (perception vs. production), while in signed language it is the specific type of neighbor (location vs. handshape) that determines facilitation and inhibition. How might we account for these differences? One possibility is to assume that there are different computational principles at work in signed and spoken language, leading to fundamental differences in the way words and signs are activated during language processing (e.g., Corina and Knapp, 2006b; Baus et al., 2008). The fact that it matters in sign language whether a neighbor shares its location or its handshape with the target suggests that there are sign language-specific retrieval mechanisms since there is no exact corollary of these parameters in spoken language. These different mechanisms could have their origins in the different neural substrates that may underlie signed and spoken word processing. For example, the difference between location and handshape in sign processing may be due to the fact that spatial location and object recognition are carried out via different neural “streams” in the visual system (e.g., Mishkin et al., 1983). The different mechanisms could also arise because handshapes are compositionally more complex than locations since they comprise many features (selected fingers, abduction, etc.) while locations can be specified by a single feature (e.g., *shoulder*; Corina and Knapp, 2006b). Another difference is that handshape is perceived categorically, while location is not (Emmorey et al., 2010). These sorts of explanations imply that the language architecture differs across the modalities.

Another possibility is that spoken and signed languages make use of the same core mechanisms to access the mental lexicon and it is a handful of relatively peripheral differences between modalities that accounts for the differences in the way neighbors affect processing. Chen and Mirman's theory of lexical access accounts for the pattern of reversals observed in spoken (and written) language with a single core lexical access mechanism, varying only the most peripheral elements across modality (the sequence of activation of sub-lexical units in speech perception and word recognition). In the same way, it could be the case that the same computational mechanism underlies sign and word processing and the pattern of reversals apparent in sign language is a result of variation in the peripheral facts about location and handshape in signs. To the point, location neighbors may be inhibitory and handshape neighbors facilitatory because facts about sign locations and handshapes may make location neighbors stronger competitors than handshape neighbors.

In the present investigation, we explore three reasons that location neighbors might generally be stronger competitors than handshape neighbors. The first possibility relates to the temporal order of a sign's perception. As a sign unfolds over time, location is identified ~30 ms earlier in perception than handshape (Grosjean, 1981; Emmorey and Corina, 1990, though see Morford and Carlson, 2011). This might mean that location sub-lexical units send activation to neighbors for a relatively long time, enabling location neighbors to become strong competitors. By the same token, the later recognition of handshape might mean that handshape sub-lexical units become activated later in time and send activation to neighbors for only a relatively short amount of time, leading handshape neighbors to become only weakly activated. It is thus possible that the timing of sub-lexical feature activation in perception is what causes location neighbors to be inhibitory and handshape neighbors to be facilitatory in recognition.

The second possibility relates to the absolute number of neighbors a target sign has. Although Carreiras et al.'s (2008) design crossed neighbor type (location/handshape) with density (high/low), the number of neighbors in the high and low density conditions varied across neighbor type. Specifically, the high density location neighborhoods were almost seven times larger on average than the high density handshape neighborhoods. It could be simply that the purported difference between location and handshape neighborhoods was actually due to the difference in neighborhood size across the location and handshape conditions. That is, it is possible that a large number of neighbors (e.g., the number of neighbors in the location condition) inhibits perception, but a “medium” amount of neighbors (e.g., the number of neighbors in the handshape condition) facilitates perception. According to this hypothesis, it is the absolute number of neighbors that causes location neighbors to be inhibitory and handshape neighbors to be facilitatory in recognition.

The last possibility is that location is more robustly represented than handshape. There is a wealth of evidence that this may be the case. Location is misperceived less frequently than other features (Orfanidou et al., 2009), and is easier to remember than movement and orientation (Thompson et al., 2005). Location errors are less frequent than handshape errors (Klima and Bellugi, 1979; Corina, 2000; Hohenberger et al., 2002), and location is learned sooner (e.g., Marentette and Mayberry, 2000). If location representations are more robust than handshape representations, location *neighbors* will become strongly activated during sign recognition while handshape neighbors will be relatively weakly activated. Within the Chen and Mirman architecture, this would cause location neighbors to have a net inhibitory effect and handshape neighbors to have a net facilitatory effect on target recognition.

There are several reasons that location may be more robustly encoded than handshape, for example, locations might be more salient, draw more attention, or be attended to at an earlier age than other sign parameters. For the purposes of this investigation, we focus on a possibility that arises because of the particular way that neighbors have been defined in sign language research. When neighbors are defined as signs that share *one* sub-lexical unit rather than signs that share all but one sub-lexical unit (as in spoken and written language research), neighborhood density is actually the same as *sub-lexical frequency*. What Carreiras et al. (2008) called an effect of neighborhood density—a lexical property—could actually be an effect of sub-lexical frequency. In their stimuli, the average location was seven times more frequent in the language than the average handshape. We consider the possibility that sub-lexical frequency (or other factors, such as salience/attention) influences how robustly sub-lexical units are encoded, which we instantiate as different levels of resting activation. According to this proposal, high frequency sub-lexical units (locations) could have high resting levels of activation leading location neighbors to become strong (inhibitory) competitors. Low frequency sub-lexical units (handshapes) could have low resting levels of activation, leading handshape neighbors to become weak competitors and result in net facilitation.

We report the results of 3 simulations of sign recognition using a lexical network that utilizes the activation principles proposed by Chen and Mirman (2012) and that incorporates differences in sub-lexical activation and timing and neighborhood density, as described above. The use of computer simulations allows us to test how sign perception could function in a system that has no intrinsic location or handshape, or any other sign-specific features. We can test whether the factors that influence the strength of a neighbor's activation described above are sufficient for obtaining the observed pattern of facilitation and inhibition. If the simulations are capable of reproducing the observed effects, they will serve as a proof of concept that language-general principles are sufficient to account for lexical access in sign language. If the simulation is incapable of reproducing the empirical results, we conclude that sign access involves different—i.e., sign language-specific—retrieval mechanisms than spoken language (though null results are always difficult to interpret).

## Model architecture

**Figure 2:**
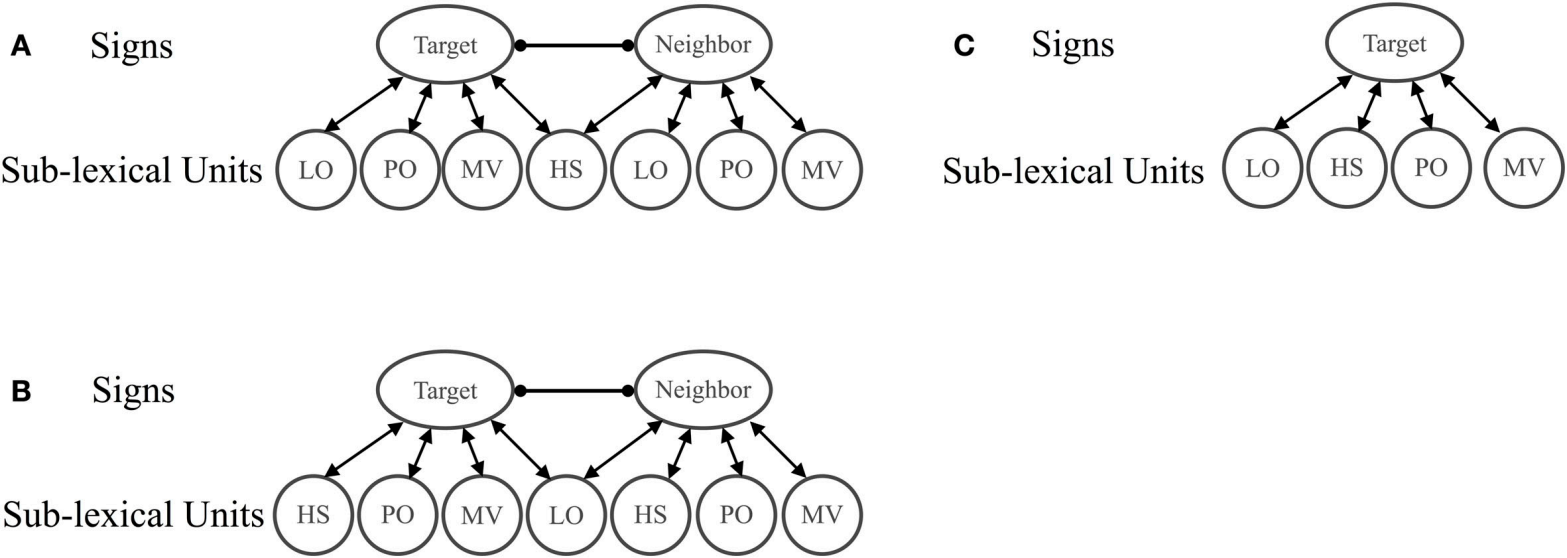
**Model Architecture**. Activation of the target with a handshape neighbor **(A)** or location neighbor **(B)** was compared to activation of the target without a neighbor **(C)**. Neighbors were considered to have a facilitatory effect on sign recognition if the target item with a neighbor **(A,B)** became active more quickly than the target item without a neighbor **(C)**. Neighbors were considered to have an inhibitory effect if the target item with a neighbor became active more slowly than the target item without a neighbor.

Like Chen and Mirman (2012), the structure of the architecture comprised two layers of units: a sub-lexical level and lexical level (see Figure 2). Bidirectional facilitatory weights connected the lexical and phonological levels, and unidirectional lateral inhibitory weights connected lexical items (see Table 1 for parameter values). As in Chen and Mirman (2012) lateral inhibitory connections were scaled by a sigmoid function of word activation that forces rapid selection of only one lexical item (in all models β = 35 and *x*_0_ = 0.3, following Chen and Mirman):

$$y=\frac{15}{1.5+e^{-\beta (x-x_{0})}}$$

**Table 1:** **Values Used in All Simulations**.

**Parameter**	**Value**
Sub-lexical unit to sign excitation	0.2
Sign to sub-lexical unit excitation	0.2
Sign to sign inhibition	See formula
Resting activation	0 unless otherwise specified
Sub-lexical unit Decay	0
Word Decay	0

In order to simulate the recognition of a single target sign, the sub-lexical units associated with the target were activated through external input, and the activation of the target sign was taken as a measure of lexical access. The simulations reported here orthogonally varied the timing (Simulation 1) and amount of activation given to the sub-lexical units (Simulation 2) as well as the number of neighbors shared by the target (Simulation 3). We provide the details of these manipulations in the simulations below. Note that we modeled average reaction times for each cell (density: high and low; neighbor type: handshape and location) rather than reaction times for particular items. The assumptions regarding timing, sub-lexical frequency, and neighborhood density were also derived from averages rather than particular lexical items. The net effect of a neighbor on the target was calculated by subtracting the activation of a target no neighbors from the activation of the target with a neighbor (or neighbors). The simulations presented here were implemented using PDPtool in MATLAB (McClelland, 2009).

## Simulation 1: timing

In Simulation 1, we tested the hypothesis that the effects of location and handshape can actually be attributed to the sequence with which sub-lexical units become active in perception. To do this, we manipulated the timing of the activation of the sub-lexical units in accordance with the average time of sub-lexical unit identification from behavioral data. Emmorey and Corina (1990) report that location and orientation are identified first (146 ms on average), followed by handshape (172 ms), and then movement (238 ms). To simulate timing, two of the target sub-lexical units (“location” and “orientation”) received input for 3 cycles (equivalent to ~30 ms) before the “handshape” sub-lexical unit was activated for 7 cycles (equivalent to ~70 ms). Finally, the “movement” sub-lexical unit was activated for the remaining cycles. The effect of having a location neighbor was simulated by creating an additional lexical unit that shared the location unit with the target but had distinct orientation, handshape, and movement features (see Figure 2A). The effect of having a handshape neighbor was simulated the same way, except that the neighbor shared the handshape unit with the target (see Figure 2B). Since we are simulating the recognition of the target item, only the target's sub-lexical units received activation—none of the neighbor's sub-lexical units were activated except for the shared unit. The amount of external input applied to the sub-lexical units was set to 2, though we explored other levels of activation and the results were qualitatively the same throughout.

### Simulation 1 results

**Figure 3:**
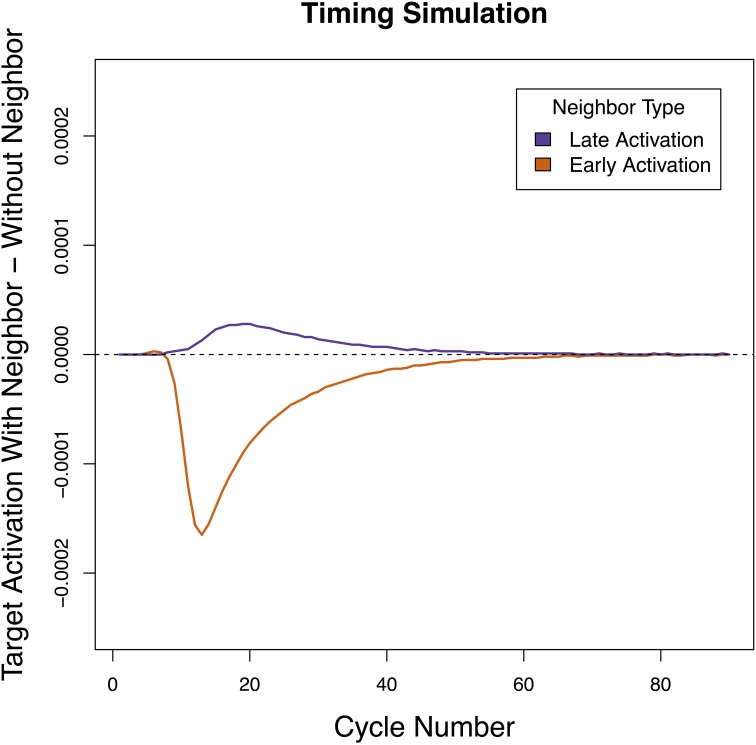
**Net contribution of a handshape neighbor and a location neighbor when the timing of sub-lexical sub-lexical unit activation was manipulated**. Handshape neighbors had a net facilitatory effect on the target, while location neighbors had a net inhibitory effect on the target.

The results of Simulation 1 are presented in Figure 3. As predicted, when the shared sub-lexical unit became active early in processing (as is empirically the case with location), the neighbor contributed net inhibition to the target sign. When it became active late in processing (as has been demonstrated for handshape), the neighbor contributed net facilitation to the target sign. The fact that the network tested in Simulation 1 produced the correct pattern of behavior suggests that the inhibition and facilitation observed for location and handshape neighbors in sign recognition may be due to differences in when different sub-lexical units are activated in perception.

## Simulation 2: sub-lexical frequency

In Simulation 2, we tested the hypothesis that the effects of location and handshape could actually be due to differences in how robustly encoded the sub-lexical units are. We simulated this possibility by manipulating the resting level of activation of the sub-lexical units in accordance with the average sub-lexical frequencies of the location and handshape parameters. As described above, in the existing behavioral research the high density location neighborhoods (*M* = 203, range = 203–203) were almost seven times larger than the high density handshape neighborhoods (*M* = 28, range = 21–35; Carreiras et al., 2008). To model this difference, the resting activation of one sub-lexical unit (the “location” unit) was set to 0.7 while the resting level of the other units was set to 0.1. The amount of external activation applied as input to the sub-lexical units was set to 1, though the results are qualitatively the same with other levels of input. All sub-lexical units received external activation simultaneously, rather than sequentially as in Simulation 1. We note that resting level of activation is only one way of modeling frequency (Dahan et al., 2001; Knobel et al., 2008), and resting activation could also be thought to correspond to attention or salience (e.g., Mirman et al., 2008).

### Simulation 2 results

**Figure 4:**
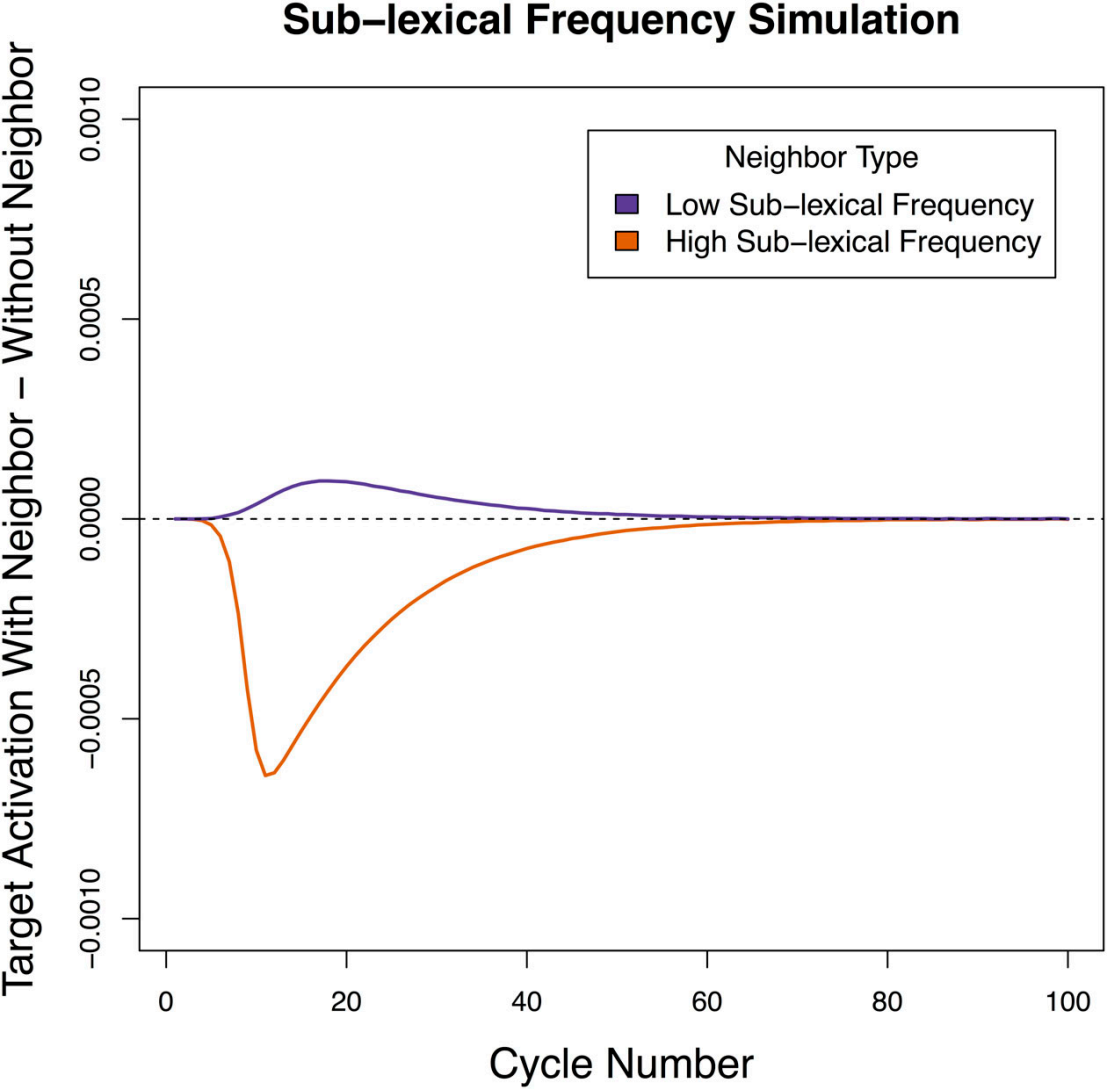
**Net contribution of a handshape neighbor and a location neighbor when the resting level of activation of sub-lexical units was manipulated**. Handshape neighbors had a net facilitatory effect on the target, while location neighbors had a net inhibitory effect on the target.

As in Simulation 1, Simulation 2 revealed that a when the shared feature had high resting activation the neighbor contributed net inhibition to the target sign, and when the shared feature had low resting activation (which corresponded to handshape) the neighbor contributed net facilitation to the target sign (see Figure 4). The results were qualitatively the same within ±0.2 units of resting activation. This suggests that facts about sub-lexical frequency could be responsible for the patterns of facilitation and inhibition in sign recognition.

## Interim discussion

Both simulations demonstrated that it is possible to model the pattern of reversals seen in behavioral studies of sign perception with minimal modifications to the architecture thought to underlie spoken language. At the sub-lexical level, varying either the timing of activation or the amount of resting activation is sufficient to produce quantitatively similar patterns to what has been observed with humans performing sign recognition. These results demonstrate that differences in the timing with which location and handshape targets are perceived and differences in the robustness with which these parameters are encoded (as modeled using sub-lexical frequency) are computationally tractable explanations for the pattern of reversals in sign language.

## Simulation 3: number of neighbors

The first two simulations evaluated whether manipulations of sub-lexical properties can produce the observed pattern of facilitation and inhibition. In Simulation 3 we consider whether the pattern of reversals is due to activity at the lexical level, in particular the number of neighbors that are active during processing.

**Figure 5:**
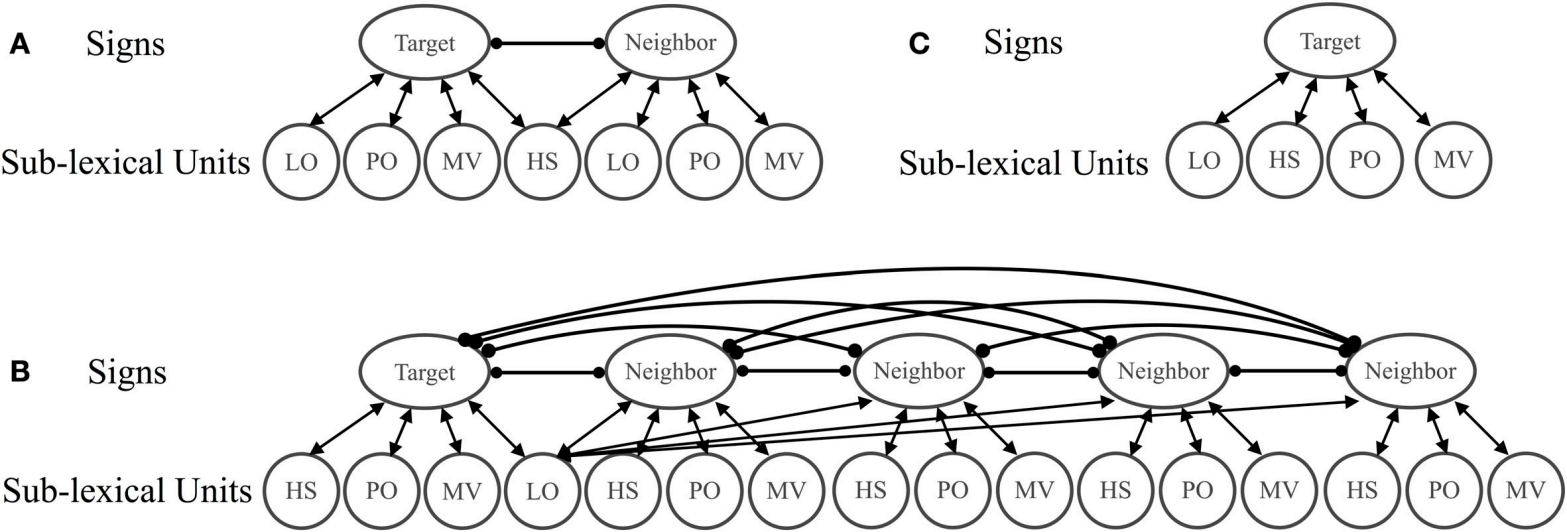
**Architecture of sign perception when Neighbor Type was manipulated by varying the number of neighbors**. Activation of the target with a handshape neighbor **(A)** or location neighbor **(B)** was compared to activation of the target without a neighbor **(C)**. Neighbors were considered facilitatory if the target item with a neighbor **(A,B)** became active more quickly than the target item without a neighbor **(C)**.

Two conditions were simulated: having a high neighborhood density (HND) and having a low neighborhood density. In the HND condition, which simulated the size of the location neighborhoods in Carreiras et al. (2008), there were four neighbors and in the low neighborhood density condition (LND; simulating the handshape neighborhoods), there was only one neighbor (see Figure 5). To determine the net contribution of the neighbor(s), the activation of the target in the LND condition (Figure 5B) and the HND condition (Figure 5A) was compared to the activation of the target without a neighbor (Figure 5C). To test the generality of the density effects, we tested LND and HND conditions using different amounts of external activation to the target sub-lexical units. We report data for external activation levels of 1 and 9 but the results are qualitatively the same at other input levels. In order to isolate the effect of lexical neighborhood density, all sub-lexical units simultaneously received the same amount of external activation.

### Simulation 3 results

**Figure 6:**
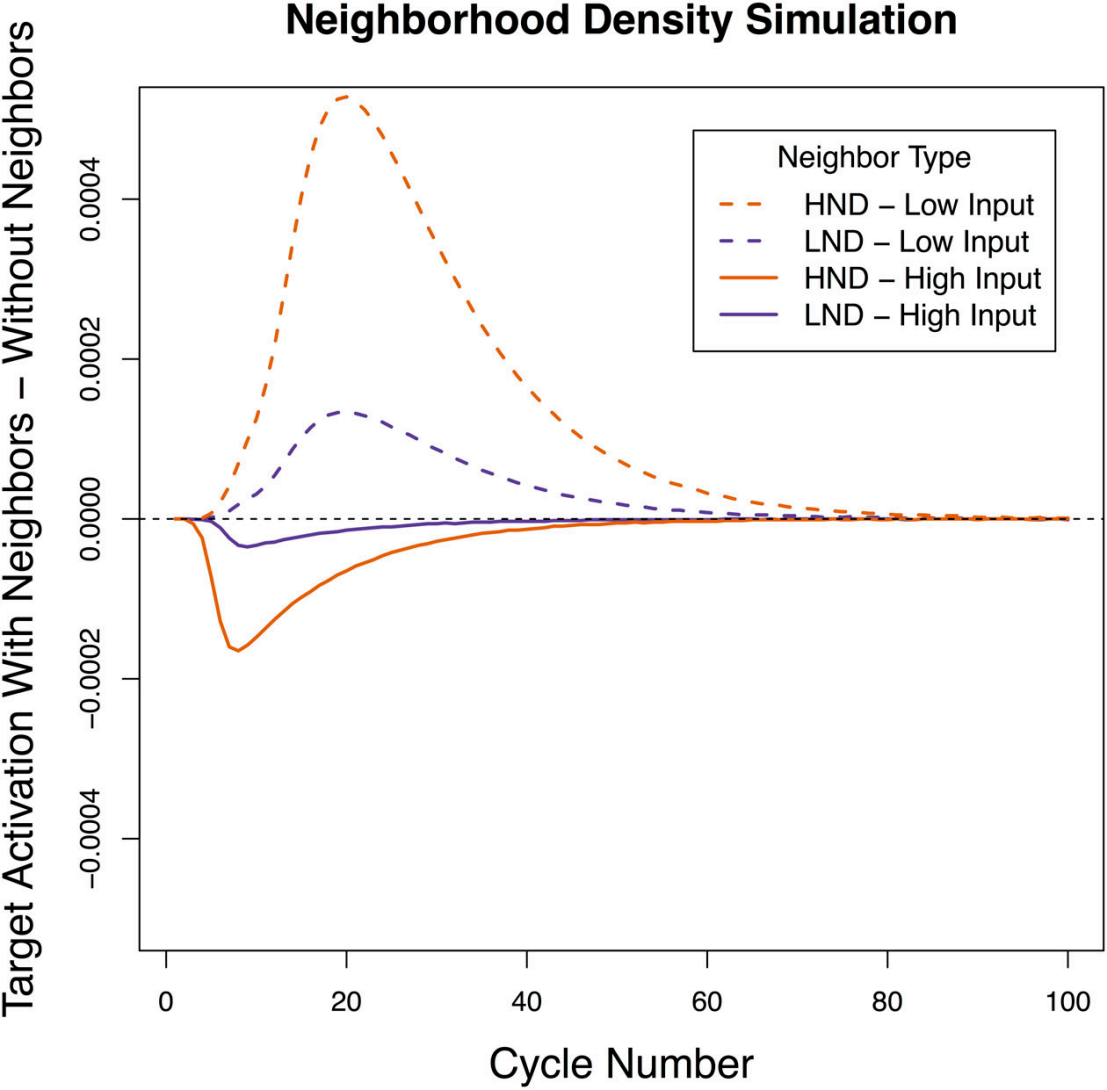
**Net contribution of a handshape neighbor and a location neighbor when the number of neighbors was manipulated**. Both high and low levels of external input are presented. Both handshape and location neighbors had a net facilitatory effect on the target when the external input was low, and both handshape and location neighbors had a net inhibitory effect on the target when the external input was high.

A very different pattern emerged in Simulation 3 than the previous 2 simulations. Here, neighborhood density did not determine the direction of the effect (the HND and LND conditions patterned together) and what determined whether the effect was facilitatory or inhibitory was the amount of activation applied to the input units (Figure 6). Specifically, when a low amount of activation was applied, both HND and LND were facilitatory and when a high amount of activation was applied, both HND and LND inhibitory. In all cases, having four neighbors magnified the effect of having a single neighbor—when a single neighbor was facilitatory, four neighbors were more facilitatory, and when a single neighbor was inhibitory, four neighbors were more inhibitory. These results suggest that the pattern of reversals linked to location and handshape in sign recognition cannot be reduced to differences in neighborhood density, a lexical property. We will discuss this pattern in more depth in the General Discussion.

## General discussion

The aim of the present study was to computationally test the hypothesis that behavioral patterns in sign recognition can be accounted for using the same lexical access mechanisms that have been proposed for spoken language. Specifically, we investigated whether the opposing effects observed for location and handshape can be obtained in a lexical network that employs universal (language-general) activation principles mediated by language-specific facts about activation levels and neighborhoods.

To do so, we created a spreading activation network with two levels of representation (sub-lexical and lexical) and two types of activation: facilitatory, bidirectional connections between sub-lexical and lexical units; and inhibitory, activation-scaled, unidirectional connections between lexical units (Chen and Mirman, 2012). We then systematically varied three relatively peripheral facts about this network: (1) the timing with which sub-lexical units become active during perception, (2) the resting activation of the sub-lexical units, and (3) the number of lexical neighbors of a target sign. These factors were orthogonally tested in a simulated recognition task with parameters drawn from empirical data about sign languages [specifically: (1) the timing of the perception of location vs. handshape, (2) the sub-lexical frequency of locations vs. handshapes, and (3) the number of a target's location neighbors vs. handshape neighbors].

We found that the specific pattern of facilitation and inhibition reported in sign recognition was obtained when the timing of sub-lexical activation (Simulation 1) and the level of sub-lexical resting activation (Simulation 2) were varied in a manner consistent with real-world facts about location and handshape. We were not able obtain the observed pattern of results when the number of lexical neighbors was similarly varied (Simulation 3). Before drawing conclusions from these results, we wish to address why the network presented a different pattern of results depending on whether sub-lexical or lexical properties were manipulated.

To understand why variations in properties of the shared sub-lexical unit (timing/resting activation) determined whether the net contribution of the neighbor was facilitatory or inhibitory but variations in the size of the lexical neighborhood did not, it is useful to return to the basic principle at the heart of Chen and Mirman (2012)'s architecture: strong neighbors inhibit target processing while weak neighbors facilitate processing. Differences in the timing and resting activation of a shared sub-lexical unit directly influence how active the neighbor becomes, which in the Chen and Mirman architecture determines whether its net contribution to the target will be negative or positive. In other words, variation in the sub-lexical properties can change the *polarity* of the activation flowing to the target from net positive to net negative. This is why the sub-lexical variations we explored in Simulations 1 and 2 led to differing patterns of facilitation and inhibition. What, then, is the effect of giving a target sign fewer or more neighbors, as in Simulation 3? The crucial fact in this case is that varying the number of neighbors a target has does not influence whether the neighbors themselves are strongly or weakly activated. Because all the neighbors in this model are activated by the same sub-lexical unit, the amount of activation they receive is the same. Therefore, whatever the effect of a single neighbor is in this model, the effect of multiple neighbors will be the same. While the neighbors will become more strongly or less strongly active based on the properties of the sub-lexical units, all of the target item's neighbors will either be net facilitatory or net inhibitory but not both. In other words, the number of neighbors thus does not change the *polarity* of the activation flowing to the target but it does influence the *magnitude*.

In this paper, we attempted to simulate a set of experimental data in order to test the theory that lexical access is accomplished by the same mechanisms in signed and spoken language. Our interpretations about the theory instantiated by the simulation necessarily depend on the assumptions made both in the creation of the simulation and in the design of the original experiments. One concern is that the definition of neighbors used by Carreiras et al. (2008) differs from what is used in research on spoken language. At the moment it is unclear which definition is most appropriate for sign processing (and across different ages of acquisition: Mayberry and Witcher, 2005) and more work is needed to decide this issue. We note, however, that the one-feature-shared definition may have more generalizability than the all-but-one-shared definition simply as there are very few minimal pairs in sign languages relative to spoken languages (van der Kooij, 2002). In addition, the behavioral data modeled here was from LSE signers. More work is needed to explore the generalizability of these results across signed languages. Lastly, the behavioral data modeled in this study consisted of only 4 datapoints from LSE: average reaction times for high vs. low location density and high vs. low handshape density (Carreiras et al., 2008). Likewise, the estimates of sub-lexical frequency and neighborhood density were also based on averages rather than particular lexical items. Future behavioral and computational work is needed to test the model using item-level (and ideally, trial-level) reaction times, sub-lexical frequency and neighborhood density estimates, and timing estimates (e.g., Balota et al., 2007), as well as to measure the goodness of fit of the model. As it stands, this work serves as a proof of concept that the same mechanism for lexical access could underlie both sign and word perception.

The goal of the work presented here was to examine a particular pattern of behavior in lexical access using a set of tightly controlled simulations. In the same way that laboratory experiments make it possible to test the effects of a small set of variables in isolation, this approach made it possible to orthogonally test the effects of neighborhood density, sub-lexical frequency, and timing. The downside of controlling simulations or experiments so tightly is that it reduces ecological validity. In humans, a number of factors—lexical familiarity (Carreiras et al., 2008) and other neighbor types (Corina and Hildebrandt, 2002; Mayberry and Witcher, 2005; Corina and Knapp, 2006a; Dye and Shih, 2006) to name two—in addition to those modeled here play a role in lexical access. We see computational modeling as an exciting tool to understand sign processing, and hope that over time models like the one presented here can be elaborated to account for many of these factors.

With these assumptions in mind, these results suggest that the pattern of reversals in sign recognition arise because of variation in the activation of sub-lexical units rather than lexical units. In particular, our simulations are consistent with the idea that the sub-lexical feature of location is more robustly encoded or activated earlier than handshape (leading to greater neighbor activation). This prediction connects nicely with other behavioral results. As was mentioned in the introduction, location is misperceived less frequently (Orfanidou et al., 2009), remembered more easily (Thompson et al., 2005), and is produced more accurately by aphasic (Corina, 2000) and unimpaired individuals (Klima and Bellugi, 1979; Hohenberger et al., 2002) than other sub-lexical features. Since activation level correlates with accuracy in spreading activation networks, these empirical results are compatible with our proposal that location representations are able to accrue more activation than handshape representations. More empirical research attempting to elucidate the locus of these various effects is certainly needed.

Our success in modeling the effects of location and handshape in Simulations 1 and 2 provides evidence that there may be universal principles governing the way the mental lexicon is accessed. Even though location and handshape are elements that are unique to sign languages, it appears that their influence on recognition can be modeled using the same principles that have been used to explain lexical access across tasks in spoken and written language. We wish to note that our results do not rule out the possibility that there are sign language-specific factors that influence lexical processing (e.g., distinct “what” vs. “where” processing streams in visual perception). They do, however, indicate that such factors are not necessary to account for the empirical data on reversals. Our investigation suggests that—like the commonalities observed in the grammars of signed and spoken languages—the mind stores and accesses words in the same manner, no matter the modality (spoken, print, or signed).

### Conflict of interest statement

The authors declare that the research was conducted in the absence of any commercial or financial relationships that could be construed as a potential conflict of interest.

## Footnotes

^1^A related reversal has been shown for semantic neighbors (words that are semantically but not phonologically related to the target). Neighbors that share many semantic features with the target inhibit processing while neighbors that share few features facilitate target processing (Mirman and Magnuson, 2008). As the simulations presented here model form (“phonological”) neighbors in sign language processing, we focus the remainder of the review on the literature in spoken word and sign language processing rather than reading or semantics.

^2^Early literature proposed 4 classes of sub-lexical units or “parameters”: handshape, location, movement, and palm orientation (Stokoe, 1972). Recently, more nuanced systems have been proposed for describing signs (Sandler, 1989; van der Hulst, 1993; Brentari, 1998; van der Kooij, 2002) though Stokoe's four parameters remain prevalent in the psycholinguistic literature.

^3^Corina and Hildebrandt (2002) found marginally significant inhibitory effects of location primes.

^4^Note that Dye and Shih (2006) found a facilitatory effect of primes that shared both movement and location. However, because targets and primes shared two sub-lexical units, it is difficult to know whether the source of the effect was location, movement, or an interaction of the two.
